# Dual Aptamers‐Based SETDB1 PROTACs as Effective Anti‐Tumor Strategies for Breast Cancer

**DOI:** 10.1002/advs.202521159

**Published:** 2026-01-07

**Authors:** Yanxuan Guo, Yingge Lv, Shuyu Huang, Chang Liu, Yan Ouyang, Bei Lan, Chenghao Xuan

**Affiliations:** ^1^ The Province and Ministry Co‐Sponsored Collaborative Innovation Center for Medical Epigenetics State Key Laboratory of Experimental Hematology Key Laboratory of Breast Cancer Prevention and Therapy (Tianjin Medical University) Ministry of Education Key Laboratory of Immune Microenvironment and Disease (Tianjin Medical University) Ministry of Education Department of Biochemistry and Molecular Biology School of Basic Medical Sciences Tianjin Medical University Tianjin China; ^2^ Hangzhou Institute of Medicine Chinese Academy of Sciences Hangzhou Zhejiang China

**Keywords:** aptamer, breast cancer, PROTAC, SETDB1

## Abstract

PROteolysis TArgeting Chimeras (PROTACs) have emerged as a promising strategy for drug development targeting oncogenic proteins. Here, we report the development and characterization of dual aptamer‐based PROTACs targeting SET domain bifurcated histone lysine methyltransferase 1 (SETDB1), a key epigenetic regulator implicated in breast cancer progression, drug resistance, and tumor immune evasion. Using the Systematic Evolution of Ligands by Exponential Enrichment (SELEX) process, we identified a high‐affinity single‐stranded DNA (ssDNA) aptamer against SETDB1. This aptamer was conjugated to the nucleolin‐targeting aptamer AS1411, generating a single‐strand PROTAC (AP‐SETDB1‐S6A) and a partial double‐strand PROTAC (AP‐SETDB1‐D2), both of which exhibit good serum stability. These two PROTACs directly penetrate breast cancer cells and effectively recruit the E3 ligase mouse double minute 2 homolog (MDM2) to SETDB1, inducing proteasome‐dependent degradation of SETDB1. Functional assays demonstrated that both AP‐SETDB1‐S6A and AP‐SETDB1‐D2 significantly inhibit breast cancer cell proliferation and migration, and resensitize drug‐resistant breast cancer cells to tamoxifen. Notably, they further enhance the cytotoxic activity of CD8^+^ T cells against breast cancer cells and directly target breast cancer cells to suppress tumor growth in vivo. This study establishes dual aptamers‐based PROTACs targeting SETDB1, offering effective therapeutic strategies for breast cancer treatment.

## Graphical abstract

This study establishes dual‐aptamer PROTACs targeting SETDB1 using a SETDB1‐specific aptamer conjugated to AS1411. The designed PROTACs penetrate cells, recruit MDM2 to degrade SETDB1, and inhibit cancer cell proliferation and migration. Remarkably, they also overcome tamoxifen resistance and enhance CD8^+^ T cell cytotoxicity, suppressing tumor growth in vivo. This strategy offers an effective therapeutic approach for breast cancer.

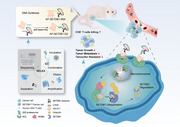

## 1 Introduction

Targeted protein degradation (TPD) technology has emerged as a powerful strategy for targeting traditionally “undruggable” proteins, significantly expanding the range of druggable targets. This innovative approach utilizes a recognition moiety that selectively binds to the target protein, facilitating its degradation through the protein hydrolysis pathway [1]. Ubiquitin‐proteasome system (UPS)‐dependent TPD approaches include molecular glue, Proteolysis Targeting Chimeras (PROTACs) [2], HaloTag [3], and dTag systems [4]. Among these, PROTACs have demonstrated particular promise due to their ability to target native, unmodified proteins that more accurately reflect pathophysiological states. PROTAC comprises three key components: a ligand for the protein of interest (POI), an E3 ubiquitin ligase ligand, and a linker that facilitates the formation of a ternary complex, ultimately leading to POI ubiquitination and subsequent proteasomal degradation  [2]. To date, PROTAC technology has been successfully applied to degrade over 100 proteins [5], with several candidates currently undergoing clinical evaluation [6, 7, 8].

The efficacy of PROTAC‐mediated protein degradation fundamentally relies on the precise recognition and binding of target proteins. In this context, aptamers, single‐stranded DNA or RNA (ssDNA or ssRNA) molecules, have emerged as powerful molecular recognition tools. These oligonucleotides are typically selected through the Systematic Evolution of Ligands by Exponential Enrichment (SELEX) process [9]. Aptamers can bind to a wide range of targets, such as small metal ions, organic molecules, proteins, viruses, cells, and so on [9]. Due to their propensity to form complementary base pairs, aptamers can fold to sophisticated secondary structures, including stem, loop, bulge, G‐quadruplex, and kissing hairpin [10], which collectively give rise to distinctive 3D conformations capable of specific molecular recognition [11]. These 3D interactions, including hydrophobic and electrostatic interactions, van der Waals forces, shape complementarity, hydrogen bonding, and base stacking drive the formation of aptamer‐target complexes [11]. This molecular complexity endows aptamers with exceptional binding affinity and specificity, enabling them to discriminate between conformational isomers [12] and even wild‐type versus mutant proteins [13]. These remarkable properties have recently been exploited in the development of aptamer‐based PROTACs.

AS1411 is a 26‐nucleotide, quadruplex‐forming ssDNA aptamer that selectively targets nucleolin (NCL) [14]. Under physiological conditions, NCL acts as a shuttle protein, facilitating the transport of ribosomal proteins from the cytoplasm to the nucleus during ribosome assembly. However, in cancer cells, NCL is frequently overexpressed and aberrantly localized on the cell surface, where it serves as a receptor for AS1411‐mediated tumor‐specific delivery and subsequent internalization of therapeutic agents [15, 16]. Notably, intracellular NCL has also been identified as an interacting partner of mouse double minute 2 homolog (MDM2) [17], a widely utilized E3 ligase in PROTAC systems. Given these properties, AS1411 presents a promising platform for PROTAC development by enabling the formation of an AS1411‐NCL‐MDM2 ternary complex [18]. AS1411‐NCL‐MDM2‐based PROTACs have been constructed to degrade STAT3, c‐Myc, p53‐R175H, AR‐V7, and c‐MET [18, 19]. These PROTACs efficiently penetrate tumor cells and degrade the POIs. The inherent hydrophilicity of aptamers also addresses the solubility limitations commonly associated with small molecule‐based PROTACs, further enhancing their therapeutic potential.

The regulation of eukaryotic gene expression involves a complex interplay of genetic and epigenetic mechanisms, including DNA, RNA, and histone modifications, chromatin remodeling, and long non‐coding RNA regulation. Among these, histone H3 lysine 9 (H3K9) methylation, catalyzed by SET domain‐containing methyltransferases, plays a pivotal role in gene silencing and heterochromatin formation [20]. SET domain bifurcated histone lysine methyltransferase 1 (SETDB1), a key enzyme in this process, primarily mediates H3K9 di‐ and tri‐methylation. SETDB1 is a critical regulator of various cellular processes, ranging from early embryonic development to immune cell maturation. Aberrant expression of SETDB1, resulting from amplification, mutation, or deletion, has been implicated in the pathogenesis of diverse diseases, including cardiovascular and gastrointestinal diseases, neurological disorders, and, most prominently, cancers [21]. Emerging evidence has established SETDB1 as an oncogenic driver in multiple malignancies, including breast cancer [22, 23], pancreatic ductal adenocarcinoma [24], melanoma [25], lung cancer [26], and hepatocellular carcinoma [27]. Furthermore, SETDB1 has been identified as a critical mediator of tumor immune evasion, highlighting its potential as a therapeutic target for restoring tumor immune surveillance [28]. These collective findings underscore the therapeutic potential of targeting SETDB1 in cancer treatment. However, research on SETDB1 inhibitors and associated structures remains in the early stage, and the development of SETDB1 antagonists is limited [21]. The bifurcated architecture of the SET domain has made it challenging to resolve the crystal structure of SETDB1 active site, therefore, most of the SETDB1 inhibitors used in preclinical testing were non‐specific antagonists [29]. Although (R, R)‐59 has been identified as the first potent and selective small‐molecule inhibitor targeting the SETDB1 tandem Tudor domains (TTDs) [30], its precise contribution to regulating SETDB1 activity across diverse substrates is still unclear [31]. Moreover, conventional small‐molecule inhibitors primarily rely on strong pocket binding and enzymatic inhibition [32], which may induce drug resistance during long‐term treatment. Therefore, PROTAC technology offers a promising strategy for generating SETDB1 antagonists by improving target specificity and achieving sustained degradation of the protein.

In the present study, we employed the SELEX methodology to isolate ssDNA aptamers with high affinity for SETDB1. The selected aptamer was conjugated to AS1411, generating a single‐strand PROTAC and a partial double‐strand PROTAC, designated AP‐SETDB1‐S6A and AP‐SETDB1‐D2, respectively, which show good serum stability. Our results indicate that AP‐SETDB1‐S6A and AP‐SETDB1‐D2 effectively recruit MDM2 to SETDB1 in breast cancer cells, inducing proteasome‐dependent degradation. These two PROTACs inhibit the proliferation and migration of breast cancer cells, and promote the sensitivity of drug‐resistant breast cancer cells to tamoxifen. They enhance the killing effect of CD8^+^ T cells on breast cancer cells and suppress the growth of breast cancer in mice. This work establishes aptamer‐based PROTACs targeting SETDB1, offering a promising strategy for breast cancer treatment and potentially paving the way for targeting other challenging oncoproteins.

## 2 Results

### 2.1 In Vitro Selection of ssDNA Aptamers for SETDB1 Protein

**FIGURE 1:**
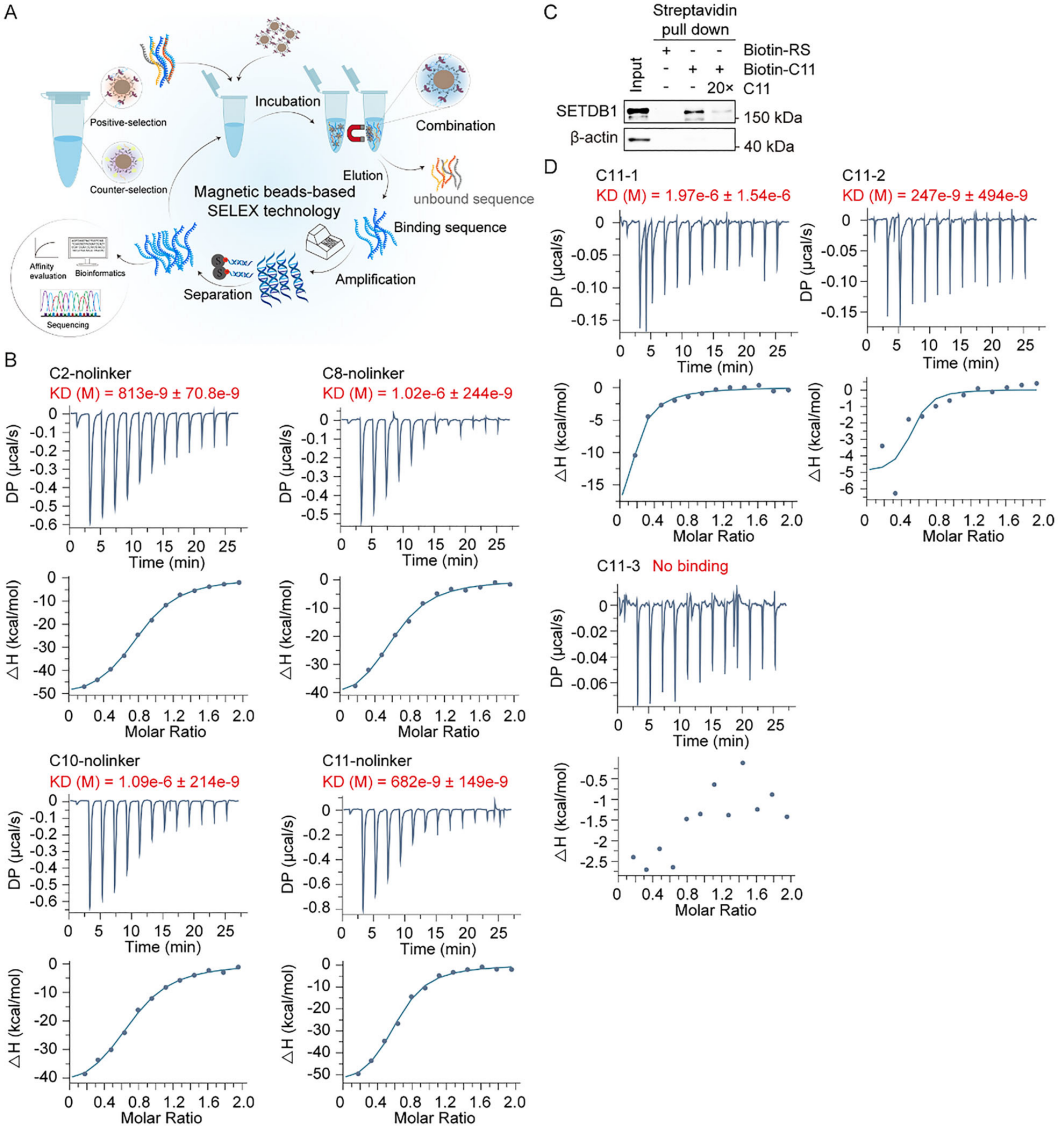
In vitro selection of ssDNA aptamers for SETDB1 protein. (A) The schematic diagram illustrating the SELEX procedure. Reproduced with permission [55] (Copyright Elsevier 2024). (B) ITC assays were performed to detect the binding affinity of SETDB1 TTDs (190–410 aa) with aptamers C2, C8, C10, and C11. (C) Biotin‐labeled random sequence (RS) and C11 were respectively incubated with MCF‐7 cell lysate overnight, followed by streptavidin pull‐down and Western blotting. (D) ITC assays were performed to assess the binding affinity of SETDB1 TTDs with aptamers C11‐1, C11‐2, and C11‐3.

SETDB1, a histone methyltransferase implicated in the progression of various cancers [22, 23, 24, 25, 26, 27] and tumor immune evasion [28], has emerged as a promising therapeutic target in cancer treatment. However, its PROTAC has not been developed. To construct an aptamer‐based PROTAC targeting SETDB1, we employed SELEX to screen ssDNA aptamers. The human SETDB1 protein comprises several evolutionarily conserved domains: SET, pre‐SET, and post‐SET domains in the C‐terminal region, three tandem Tudor domains (TTDs), a methyl‐CpG‐binding domain (MBD), as well as two nuclear export signals (NES) domains and two nuclear localization signal (NLS) domains in the N‐terminal region. For SELEX, we utilized a recombinant SETDB1 fragment (aa 190–410) encompassing the TTDs as the target because this fragment is the only domain in SETDB1 protein with a clear crystal structure reported [33]. The selection process is illustrated in Figure 1A. We initiated screening with an ssDNA library with a central 36‐nt random sequence flanked by fixed primer regions (5’‐TTCAGCACTCCACGCATAGC and CCTATGCGTGCTACCGTGAA‐3’). Following multiple rounds of selection, high‐throughput sequencing was performed, and the specificity coefficients of the top 100 sequences were analyzed (Figure S1A,B). Twelve enriched sequences with high specificity were synthesized and evaluated for SETDB1‐TTDs affinity using surface plasmon resonance (SPR). Four sequences (C2‐linker, C8‐linker, C10‐linker, and C11‐linker) exhibited strong binding (Figure S1C,D). Further truncation of their flanking primer regions yielded C2, C8, C10, and C11, which were subjected to isothermal titration calorimetry (ITC). The measured dissociation constants (Kd) were 0.813 µm (C2), 1.02 µm (C8), 1.09 µm (C9), and 0.682 µm (C11), confirming C11 as the highest‐affinity aptamer for SETDB1 TTDs (Figure 1B).

To validate the interaction between C11 and SETDB1 in a cellular context, we performed streptavidin pull‐down assays by incubating biotin‐labeled C11 or control random sequences with MCF‐7 cell lysate. The results demonstrated that endogenous SETDB1 was specifically captured by biotin‐labeled C11 (Figure 1C), while excess unlabeled C11 competitively attenuated this binding, confirming the specificity of the interaction between C11 and endogenous SETDB1 (Figure 1C).

Next, we systematically truncated C11 to identify the minimal functional sequence for binding to SETDB1. While C11‐1 (32 nt, delete 2 nt at each end of C11 ssDNA, Kd = 1.97 µm) retained binding, C11‐2 (28 nt, delete 4 nt at each end of C11, Kd = 0.247 µm) showed enhanced affinity. Further truncation to C11‐3 (24 nt, delete 6 nt at each end of C11) abolished binding entirely (Figure 1D), delineating C11‐2 as the optimal, compact aptamer for SETDB1.

### 2.2 Synthesis and Characterization of an Effective Aptamer‐Based ssDNA PROTAC Targeting SETDB1

**FIGURE 2:**
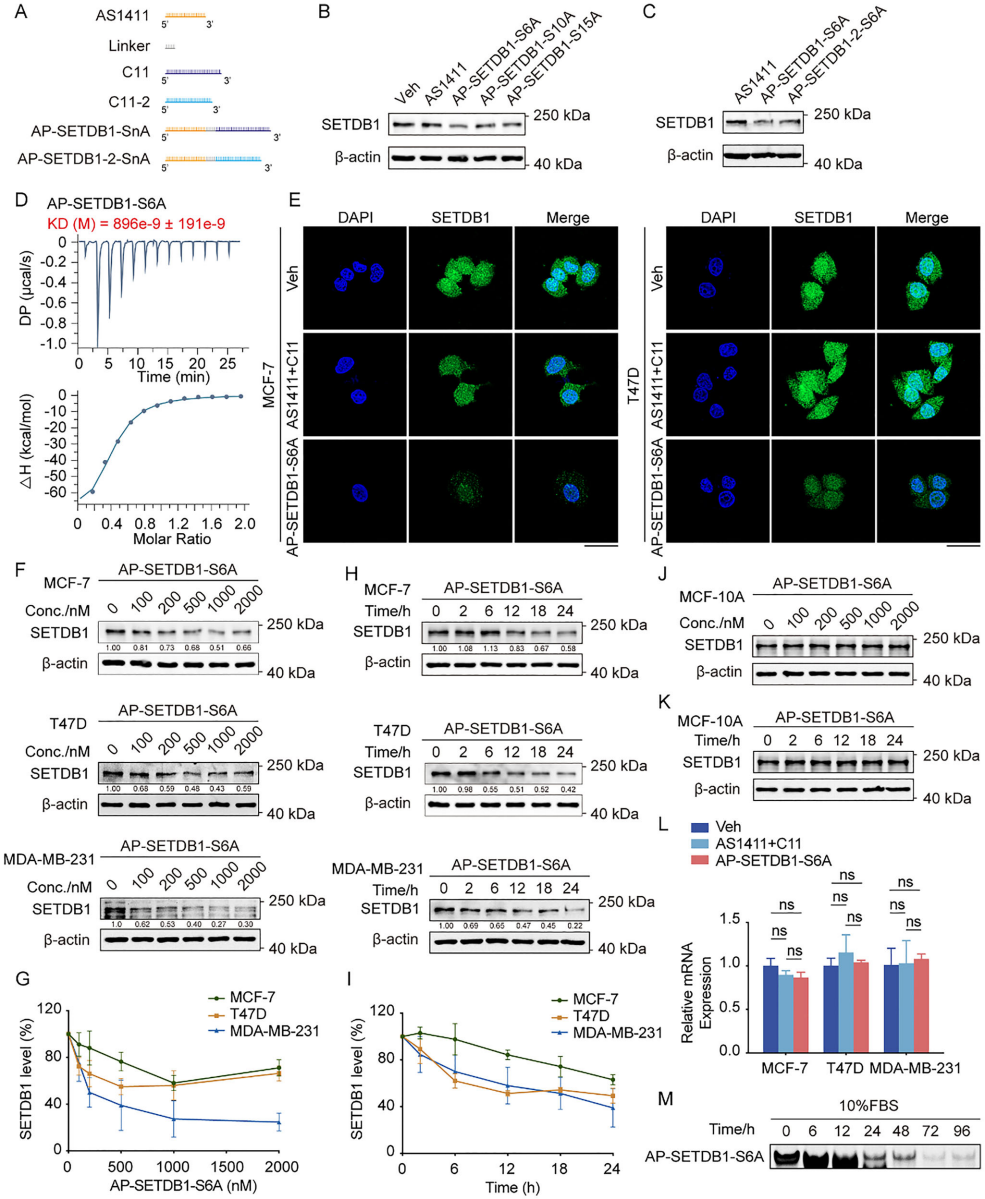
Synthesis and characterization of an effective aptamer‐based ssDNA PROTAC targeting SETDB1. (A) Schematic diagram of different ssDNA PROTACs synthesis. A series of PROTACs with poly‐adenine (A) linkers of varying lengths between AS1411 and C11 were synthesized. (B) AP‐SETDB1‐SnA PROTACs with 6, 10, or 15A as linkers were synthesized, and then incubated with MCF‐7 cells at a concentration of 1 µm for 24 h. Subsequently, cell lysates were subjected to Western blotting. (C) AS1411, AP‐SETDB1‐S6A, and AP‐SETDB1‐2‐S6A were incubated with MCF‐7 cells at a concentration of 1 µM for 24 h. SETDB1 protein levels were analyzed by Western blotting. (D) ITC assays were performed to assess the binding affinity of SETDB1 TTDs with AP‐SETDB1‐S6A. (E) MCF‐7 and T47D cells expressing GFP‐SETDB1 were incubated with 1 µm AP‐SETDB1‐S6A or a mixture of AS1411 and C11 for 24 h, and then observed under a fluorescence microscope. (F,G) MCF‐7, T47D, and MDA‐MB‐231 cells were incubated with indicated doses (0, 100, 200, 500, 1000, and 2000 nm) of AP‐SETDB1‐S6A for 24 h, and the protein level of SETDB1 was analyzed by Western blotting (F). The remaining SETDB1 was quantified (G), and each bar represents the mean ± SD for *n* = 3. (H,I) MCF‐7, T47D, and MDA‐MB‐231 cells were incubated with 1 µM AP‐SETDB1‐S6A for the indicated times (0, 2, 6, 12, 18, and 24 h), and the protein level of SETDB1 was analyzed by Western blotting (H). The remaining SETDB1 was quantified (I), and each bar represents the mean ± SD for *n* = 3. (J) MCF‐10A cells were incubated with indicated doses (0, 100, 200, 500, 1000, and 2000 nm) of AP‐SETDB1‐S6A for 24 h, and the protein level of SETDB1 was analyzed by Western blotting. (K) MCF‐10A cells were incubated with 1 µm AP‐SETDB1‐S6A for the indicated times (0, 2, 6, 12, 18, and 24 h), and the protein level of SETDB1 was analyzed by Western blotting. (L) Total mRNA from MCF‐7, T47D, and MDA‐MB‐231 cells incubating with 1 µm AP‐SETDB1‐S6A for 24 h was extracted, and real‐time quantitative RT‐qPCR assays were performed. Each bar represents the mean ± SD for *n* = 3; ns, no significant (one‐way ANOVA followed by Bonferroni post‐hoc test or Tambane's T2 post‐hoc test). (M) AP‐SETDB1‐S6A (5 µm) was incubated with the complete culture medium containing 10% FBS for the indicated times and then subjected to native PAGE.

As stated above, AS1411 has been reported to act as an indirect E3 ligand for PROTAC development through the formation of an AS1411‐NCL‐MDM2 ternary complex [18]. To develop an aptamer‐based PROTAC targeting SETDB1, we sequentially synthesized AS1411 and C11 aptamers in an ssDNA (Table S1). Given the pivotal role of linker composition in PROTAC efficacy, we engineered a series of constructs with poly‐adenine (A) as linkers with varying lengths between AS1411 and C11 (Figure 2A). These synthesized ssDNA PROTACs were directly added into the culture medium of MCF‐7 cells, and after 24 h, cellular SETDB1 protein levels were assessed by Western blotting. Among the tested PROTAC molecules, AP‐SETDB1‐S6A (aptamer‐based ssDNA PROTAC targeting SETDB1 with 6A as the linker) exhibited the most potent SETDB1 degradation activity (Figure 2B).

We also generated AP‐SETDB1‐2‐S6A using the higher‐affinity C11‐2 aptamer variant (Figure 2A). Intriguingly, despite C11‐2's superior binding affinity (Kd = 0.247 µm), AP‐SETDB1‐S6A demonstrated more effective SETDB1 degradation, suggesting potential steric constraints in the C11‐2‐based ssDNA PROTAC (Figure 2C).

Then, ITC experiments confirmed that AP‐SETDB1‐S6A maintained strong binding to SETDB1 TTDs (Kd = 0.896 µm), indicating that conjugation to AS1411 did not compromise the binding capacity of C11 aptamer to SETDB1 (Figure 2D). Further Western blotting in MCF‐7, T47D, and MDA‐MB‐231 cells established that SETDB1 degradation required the intact PROTAC construct (AP‐SETDB1‐S6A), as neither individual component (AS1411 or C11) nor their combination had the degradation effect (Figure S2). Furthermore, MCF‐7 and T47D cells, which were transfected with GFP‐SETDB1 expression constructs were treated with PBS, AP‐SETDB1‐S6A, or AS1411 together with C11, respectively. The results indicated that only AP‐SETDB1‐S6A induced the degradation of GFP‐SETDB1 in cells (Figure 2E).

Moreover, AP‐SETDB1‐S6A was demonstrated to degrade SETDB1 in MCF‐7, T47D, and MDA‐MB‐231 cells in a dose‐dependent manner from 100 to 500 nm (Figure 2F,G). However, the hook effect also occurred when the concentration of AP‐SETDB1‐S6A reached 2000 nm (Figure 2F,G). To examine the kinetics of SETDB1 degradation, we performed a time‐course experiment and detected significant degradation of SETDB1, which persisted until 24 h (Figure 2H,I). Notably, AP‐SETDB1‐S6A exhibited cancer cell‐selective activity, showing minimal effect on SETDB1 levels in non‐tumorigenic MCF‐10A cells (Figure 2J,K). This is because NCL is frequently overexpressed and aberrantly localized on the surface of cancer cells, but not normal cells [34, 35, 36, 37].

To exclude the possibility that the decrease in SETDB1 protein level after the addition of AP‐SETDB1‐S6A is caused by its transcriptional decline, we conducted real‐time quantitative RT‐PCR experiments after treatment with AP‐SETDB1‐S6A and controls. The results showed that AP‐SETDB1‐S6A treatment did not affect the transcription of SETDB1 (Figure 2L). The stability of oligonucleotide‐based therapeutics is a major concern due to their susceptibility to nuclease degradation. To evaluate the stability of AP‐SETDB1‐S6A in biologically relevant conditions, they were incubated in culture medium containing 10% fresh serum for up to 96 h and analyzed by native PAGE electrophoresis. The results revealed that AP‐SETDB1‐S6A remained stable in 48 h (Figure 2M), indicating good stability.

These collective findings establish AP‐SETDB1‐S6A as a potent, selective degrader of SETDB1.

### 2.3 Synthesis and Characterization of an Aptamer‐Based Partial dsDNA PROTAC Targeting SETDB1

**FIGURE 3:**
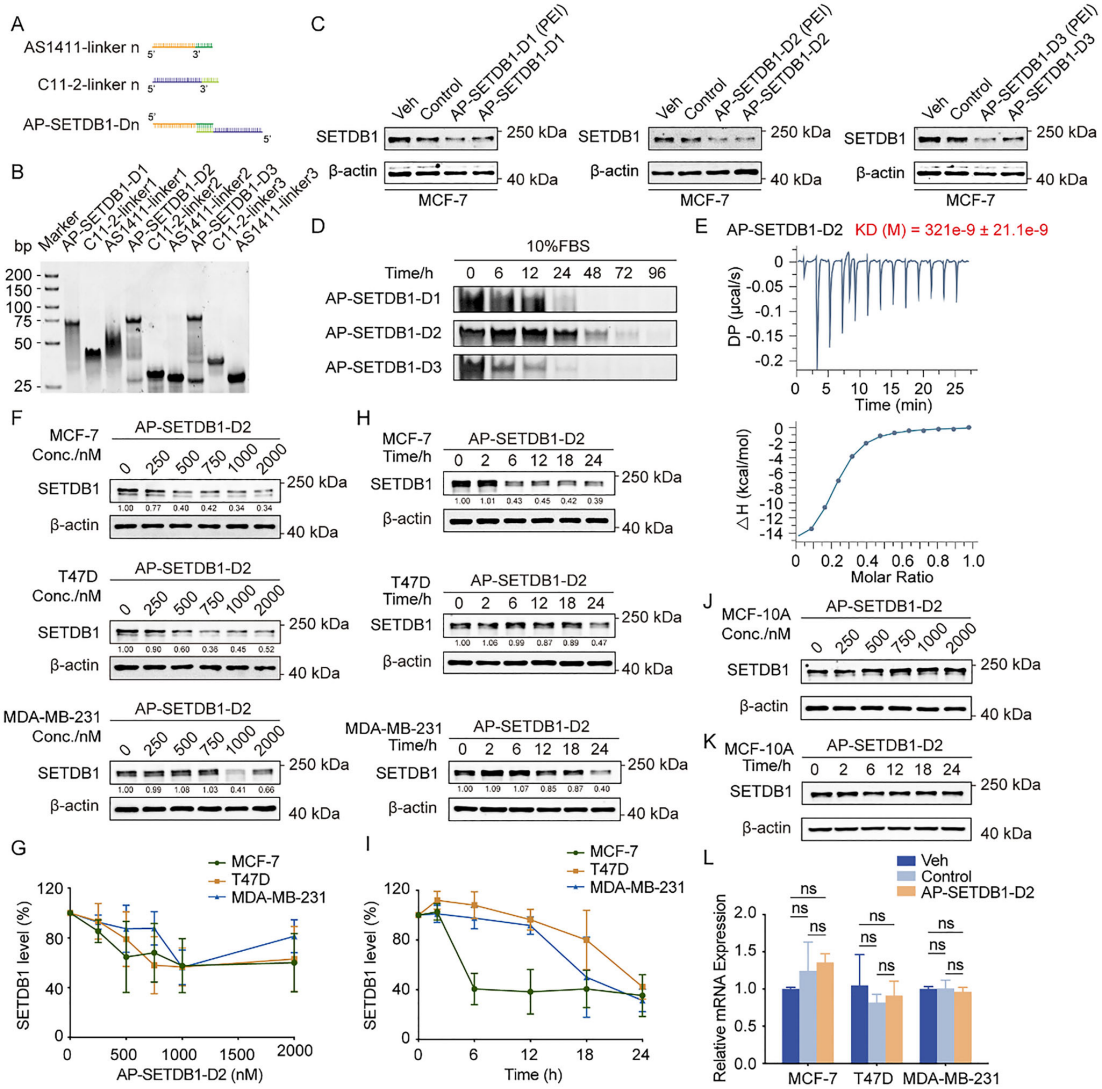
Synthesis and characterization of an aptamer‐based partial dsDNA PROTAC targeting SETDB1.(A) Schematic diagram of double‐stranded DNA linker‐based PROTAC. Dn represents different linker sequences. (B) AP‐SETDB1‐Dn was formed by connecting C11‐linker‐n and AS1411‐linker‐n through denaturation at 95°C for 10 min and then cooling to 4°C. The formation of these PROTACs were detected by native PAGE. (C) AP‐SETDB1‐D1, AP‐SETDB1‐D2, AP‐SETDB1‐D3, and control PROTAC (random DNA sequences linked to AS1411) were transfected into MCF7 cells using PEI or directly incubated with MCF‐7 cells for 24 h. The protein level of SETDB1 was analyzed by Western blotting. (D) AP‐SETDB1‐Dn (5 µm) was incubated with the complete culture medium containing 10% FBS for the indicated times and then subjected to native PAGE. (E) ITC assays were performed to assess the binding affinity of SETDB1 TTDs with AP‐SETDB1‐D2. (F,G) MCF‐7, T47D, and MDA‐MB‐231 cells were incubated with indicated doses (0, 250, 500, 750, 1000, and 2000 nm) of AP‐SETDB1‐D2 for 24 h, and the protein level of SETDB1 was analyzed by Western blotting (F). The remaining SETDB1 was quantified (G), and each bar represents the mean ± SD for *n* = 3. (H,I) MCF‐7, T47D, and MDA‐MB‐231 cells were incubated with 1 µm AP‐SETDB1‐D2 for the indicated times (0, 2, 6, 12, 18, and 24 h), and the protein level of SETDB1 was analyzed by Western blotting. (H). The remaining SETDB1 was quantified (I), and each bar represents the mean ± SD for *n* = 3. (J) MCF‐10A cells were incubated with indicated doses (0, 250, 500, 750, 1000, and 2000 nm) of AP‐SETDB1‐D2 for 24 h, and the protein level of SETDB1 was analyzed by Western blotting. (K) MCF‐10A cells were incubated with 1 µm AP‐SETDB1‐D2 for the indicated times (0, 2, 6, 12, 18, and 24 h), and the protein level of SETDB1 was analyzed by Western blotting. (L) Total mRNA from MCF‐7, T47D, and MDA‐MB‐231 cells incubating with 1 µm AP‐SETDB1‐D2 for 24 h was extracted, and real‐time quantitative RT‐qPCR assays were performed. Each bar represents the mean ± SD for *n* = 3; ns, no significant (one‐way ANOVA followed by Bonferroni post‐hoc test or Tambane's T2 post‐hoc test).

Building upon our ssDNA PROTAC design, we engineered a novel class of partial double‐stranded DNA (dsDNA) PROTACs by exploiting the intrinsic self‐assembly properties of nucleic acid aptamers. Using AS1411 and the high‐affinity C11‐2 aptamer, we constructed three distinct PROTAC variants (AP‐SETDB1‐D1, ‐D2, and ‐D3) incorporating different 10‐bp linker sequences, which were used for DNA tetrahedron formation previously [38] (Figure 3A; Table S1). Native PAGE analysis confirmed successful heterodimer formation for all three PROTACs (Figure 3B).

These three PROTACs effectively degraded endogenous SETDB1 in MCF‐7 cells, whether delivered via PEI transfection or through direct addition into culture medium (Figure 3C). The stability of AP‐SETDB1‐Dns in culture medium containing 10% fresh serum was also examined. The results revealed that AP‐SETDB1‐D2 was stable in 48 h (Figure 3D), better than AP‐SETDB1‐D1 and AP‐SETDB1‐D3. Therefore, we selected AP‐SETDB1‐D2, which had the best stability in culture medium and showed superior degradation activity when added directly to the culture medium for detailed characterization. This is because directly adding AP‐SETDB1‐D2 to the culture medium enables it to enter cells and degrade SETDB1, providing significant practical advantages by eliminating the need for additional transfection reagents. ITC measurements confirmed maintained SETDB1 binding activity, with AP‐SETDB1‐D2 exhibiting enhanced binding affinity (Kd = 0.321 µm) to SETDB1 TTDs (Figure 3E), compared to SETDB1 ssDNA PROTAC, AP‐SETDB1‐S6A (Figure 2D).

Comprehensive profiling revealed that AP‐SETDB1‐D2 mediated dose‐ and time‐dependent SETDB1 degradation across multiple breast cancer cell lines (MCF‐7, T47D, and MDA‐MB‐231) (Figure 3F–I), while it did not significantly affect the protein level of SETDB1 in non‐tumorigenic MCF‐10A cells (Figure 3J–K). The results of real‐time quantitative RT‐PCR showed that the addition of AP‐SETDB1‐D2 did not affect the transcription of SETDB1 (Figure 3L). These collective findings establish AP‐SETDB1‐D2 as a robust degrader of SETDB1.

### 2.4 AP‐SETDB1‐S6A and AP‐SETDB1‐D2 Induce SETDB1 Degradation via the Ubiquitin‐Proteasome Pathway

**FIGURE 4:**
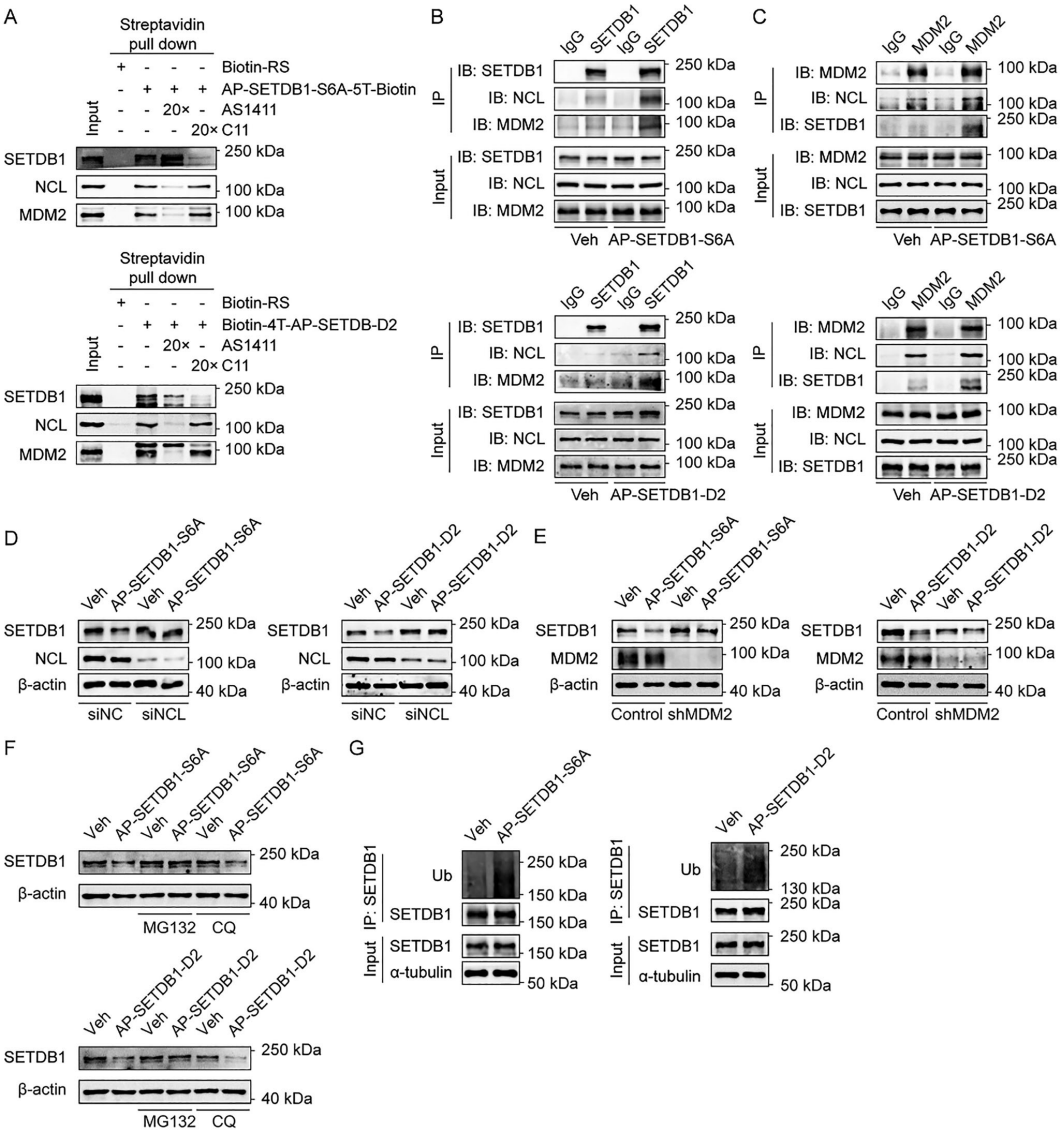
AP‐SETDB1‐S6A and AP‐SETDB1‐D2 induce SETDB1 degradation via the ubiquitin‐proteasome pathway. (A) Biotin‐labeled RS, AP‐SETDB1‐S6A, and AP‐SETDB1‐D2 were incubated overnight with MCF‐7 cell lysate supplemented with AS1411, C11, or not, followed by streptavidin pull‐down and Western blotting. (B,C) MCF‐7 cells were incubated with Veh (PBS), 1 µm AP‐SETDB1‐S6A, or 1 µm AP‐SETDB1‐D2 for 24 h, and co‐treated with MG132 (10 µm) for the last 6 h. Co‐Immunoprecipitation assays were then carried out with normal IgG, anti‐SETDB1, or anti‐MDM2, followed by Western blotting. (D) Control and NCL siRNA were transfected into MCF‐7 cells, and then the cells were incubated with Veh, 1 µm AP‐SETDB1‐S6A, or 1 µm AP‐SETDB1‐D2 for 24 h. The protein level of SETDB1 was analyzed by Western blotting. (E) MCF‐7 cells stably expressing control or MDM2 shRNA were incubated with Veh, 1 µm AP‐SETDB1‐S6A, or 1 µm AP‐SETDB1‐D2 for 24 h. The protein level of SETDB1 was analyzed by Western blotting. (F) MCF‐7 cells were incubated with Veh, 1 µm AP‐SETDB1‐S6A, or 1 µm AP‐SETDB1‐D2 for 24 h, together with MG132 or CQ (10 µm) treatment for the last 6 h. The protein level of SETDB1 was analyzed by Western blotting. (G) MCF‐7 cells were incubated with Veh, 1 µM AP‐SETDB1‐S6A, or 1 µm AP‐SETDB1‐D2 for 24 h, together with MG132 (10 µm) treatment for the last 6 h. Subsequently, cell lysates were subjected to immunoprecipitation using anti‐SETDB1, followed by Western blotting using anti‐Ub.

The efficacy of PROTACs relies on the formation of a ternary E3‐PROTAC‐POI complex. To elucidate the mechanism of SETDB1 degradation by our aptamer‐PROTACs, we first performed streptavidin pull‐down assays using biotin‐labeled AP‐SETDB1‐S6A and AP‐SETDB1‐D2. The results demonstrated that both constructs effectively co‐precipitated endogenous SETDB1, NCL, and MDM2 from MCF‐7 cell lysate (Figure 4A), with binding being competitively inhibited by excess unlabeled C11 or AS1411 (Figure 4A).

Furthermore, AP‐SETDB1‐S6A, AP‐SETDB1‐D2, or control PROTAC were added into the culture medium of MCF‐7 cells for 24 h under MG132 treatment, and then endogenous co‐immunoprecipitation assays were performed using anti‐SETDB1, anti‐MDM2, or normal IgG, followed by Western blotting (Figure 4B,C). These results showed that the addition of AP‐SETDB1‐S6A and AP‐SETDB1‐D2 promoted the interaction between SETDB1 and NCL‐MDM2 complex in cells (Figure 4B,C). Moreover, the NCL‐MDM2 dependency of AP‐SETDB1‐S6A and AP‐SETDB1‐D2‐mediated degradation was validated by siRNA/shRNA‐mediated NCL and MDM2 knockdown, both of which significantly attenuated SETDB1 degradation (Figure 4D,E).

To determine whether AP‐SETDB1‐S6A and AP‐SETDB1‐D2 degraded SETDB1 through the ubiquitin‐proteasome system, we treated cells with these two PROTACs together with the proteasome inhibitor MG132 or the autophagy inhibitor CQ. We found that MG132, but not CQ, completely blocked AP‐SETDB1‐S6A and AP‐SETDB1‐D2‐mediated degradation of SETDB1 (Figure 4F). Consistent with this finding, the results of co‐immunoprecipitation assays demonstrated that AP‐SETDB1‐S6A and AP‐SETDB1‐D2 markedly enhanced the ubiquitination of SETDB1 in MCF‐7 cells in the presence of MG132 (Figure 4G).

These results collectively demonstrate that AP‐SETDB1‐S6A and AP‐SETDB1‐D2 mediate targeted SETDB1 degradation through NCL‐MDM2‐dependent ubiquitination and subsequent proteasomal degradation.

### 2.5 AP‐SETDB1‐S6A and AP‐SETDB1‐D2 Exhibit Anti‐Tumor Activity in Cells

**FIGURE 5:**
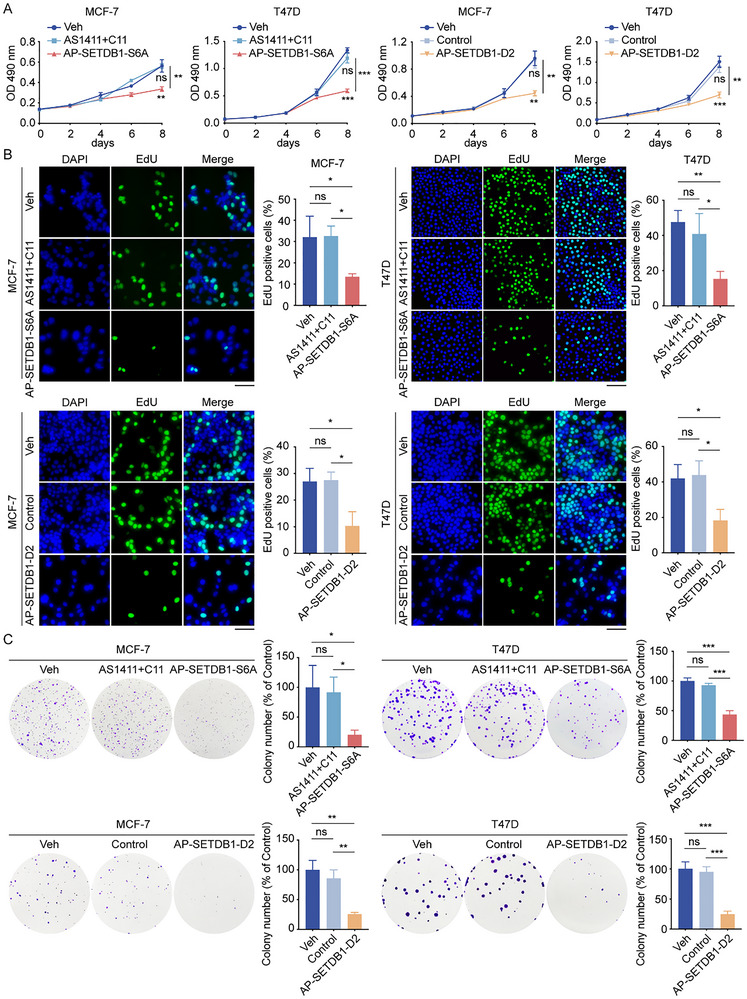
AP‐SETDB1‐S6A and AP‐SETDB1‐D2 inhibit the proliferation of breast cancer cells in vitro. (A) MTT assays were performed in MCF‐7 and T47D cells incubated with Veh, a mixture of AS1411 and C11, AP‐SETDB1‐S6A, Control dsDNA PROTAC, or AP‐SETDB1‐D2 (1 µm). (B) EdU‐incorporation assays were conducted in MCF‐7 and T47D cells incubated with indicated PROTACs for 48 h. The percentage of EdU‐positive cells was quantified. Scale bar, 100 µm. (C) Colony formation assays were performed in the indicated MCF‐7 and T47D cells. The number of colonies was counted. In Figure A–C, each bar represents the mean ± SD for *n* = 3; ns, no significant, ^*^ *p* < 0.05, ^**^ *p* < 0.01, ^***^ *p* < 0.001 (one‐way ANOVA followed by Bonferroni post‐hoc test or Tambane's T2 post‐hoc test).

Having established AP‐SETDB1‐S6A and AP‐SETDB1‐D2 as effective SETDB1 degraders in cells, we further evaluated their anti‐tumor effect in breast cancer cells. MTT assays revealed that AP‐SETDB1‐S6A and AP‐SETDB1‐D2 significantly reduced the viability of MCF‐7 and T47D cells (Figure 5A). Moreover, their anti‐proliferative effect was further confirmed by EdU‐incorporation assays, which showed decreased DNA synthesis in SETDB1‐PROTAC‐treated cells (Figure 5B). In addition, the colony formation capacity of MCF‐7 and T47D cells was also reduced by these two PROTACs (Figure 5C).

**FIGURE 6:**
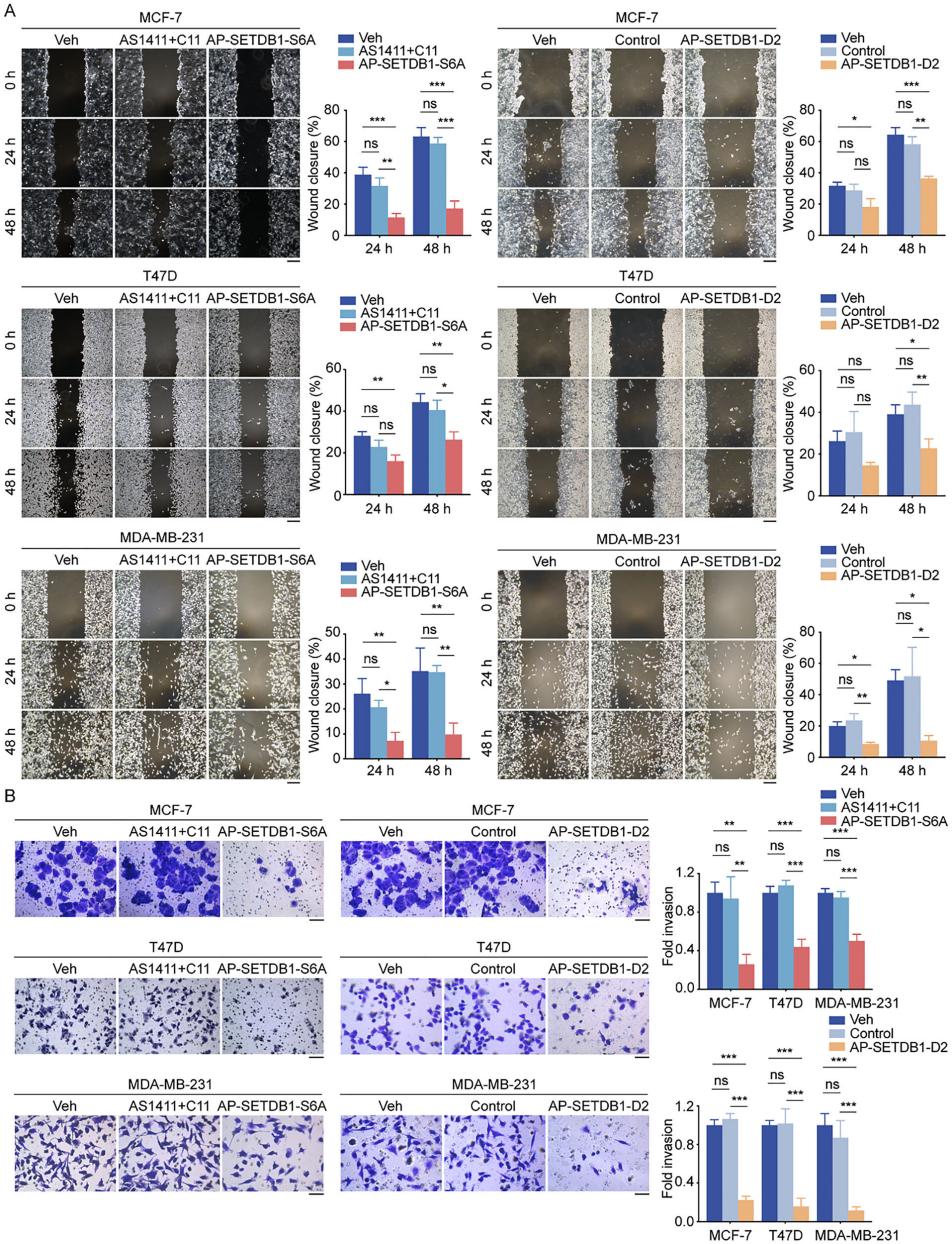
AP‐SETDB1‐S6A and AP‐SETDB1‐D2 suppress the migration and invasion of breast cancer cells in vitro. (A) Wound‐healing assays were performed in MCF‐7, T47D, and MDA‐MB‐231 cells with the indicated treatment. Scale bar, 200 µm. (B) Transwell assays were conducted in T47D and MDA‐MB‐231 cells with the indicated treatment for 24 h. Scale bar, 50 µm. In Figures A and B, each bar represents the mean ± SD for *n* = 3; ns, no significant, ^*^ *p* < 0.05, ^**^ *p* < 0.01, ^***^ *p* < 0.001 (one‐way ANOVA followed by Bonferroni post‐hoc test or Tambane's T2 post‐hoc test).

To further assess the impact of AP‐SETDB1‐S6A and AP‐SETDB1‐D2 on breast cancer cell migration and invasion ability, wound healing and transwell assays were performed. The results revealed that AP‐SETDB1‐S6A and AP‐SETDB1‐D2 remarkably attenuated the migration and invasion ability of breast cancer cells (Figure 6A,B).

**FIGURE 7:**
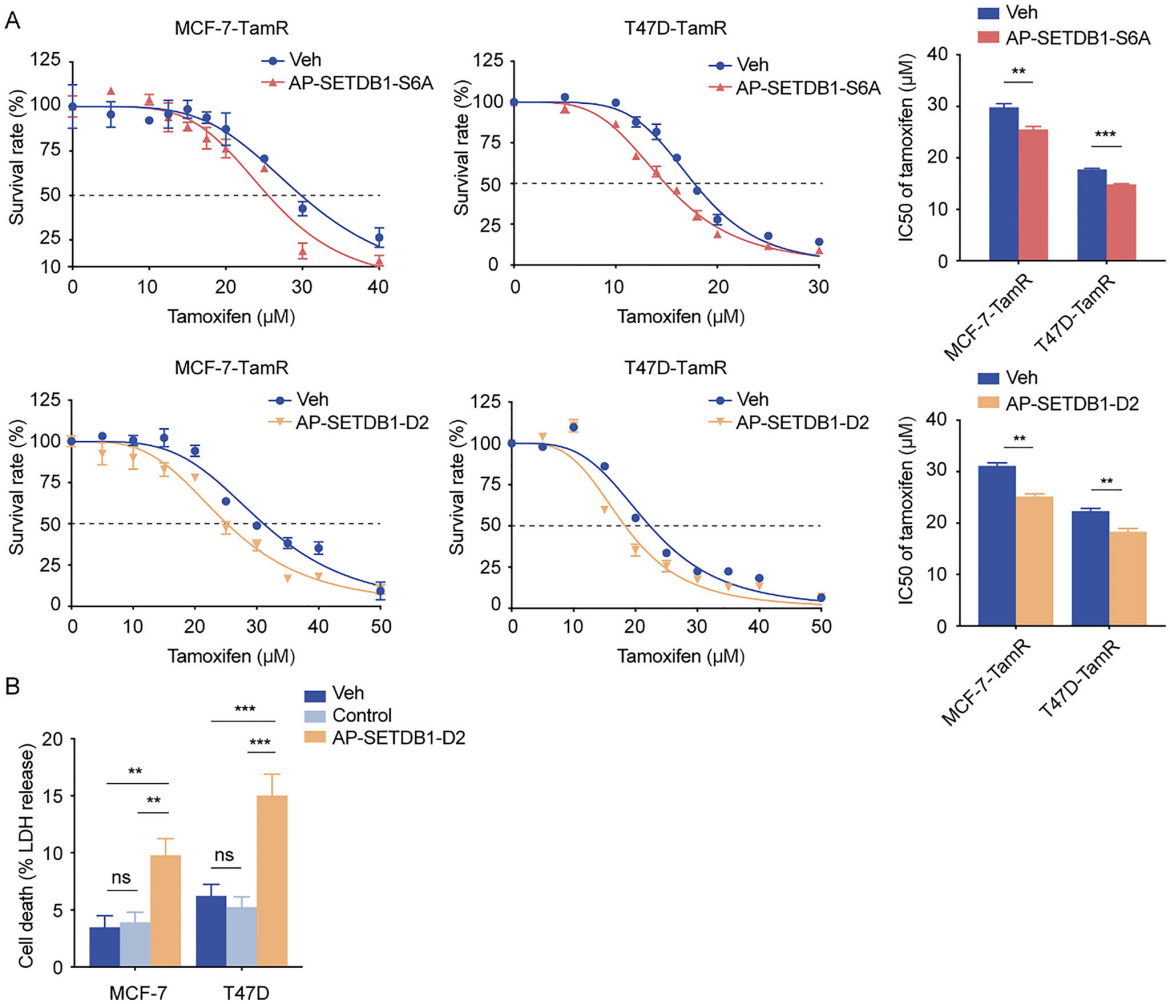
AP‐SETDB1‐S6A and AP‐SETDB1‐D2 increase the drug sensitivity of tamoxifen‐resistant cells and promote CD8^+^ T cell killing. A. MCF‐7‐TamR and T47D‐TamR cells were incubated with Veh, 1 µm AP‐SETDB1‐S6A, or 1 µm AP‐SETDB1‐D2 for 24 h, and then treated with escalating doses of tamoxifen for 48 h. The sensitivity of tamoxifen‐resistant breast cancer cells to tamoxifen was assessed using MTT assays. Each bar represents the mean ± SEM for *n* = 3; ^**^ *p* < 0.01, ^***^ *p* < 0.001 (Student's *t*‐test). (B) Human CD8^+^ T cells were isolated from human peripheral blood and activated. MCF‐7 and T47D cells were incubated with Veh, Control PROTAC, or AP‐SETDB1‐D2 (1 µm) for 48 h. Tumor cells were then co‐incubated with human CD8^+^ T cells for 48 h, and the killing ability of human CD8^+^ T cells was detected by LDH release assays. Each bar represents the mean ± SD for *n* = 3; ns, no significant, ^**^ *p* < 0.01, ^***^ *p* < 0.001 (one‐way ANOVA followed by Bonferroni post‐hoc test or Tambane's T2 post‐hoc test).

SETDB1 has been reported to participate in breast cancer cell resistance to tamoxifen [39]. Through continuous treatment with low‐dose tamoxifen, we obtained two tamoxifen‐resistant estrogen receptor‐positive breast cancer cells, MCF‐7‐TamR and T47D‐TamR. Our results of MTT assays discovered that AP‐SETDB1‐S6A and AP‐SETDB1‐D2 significantly reduced the IC50 of MCF‐7‐TamR and T47D‐TamR for tamoxifen (Figure 7A), suggesting their potential utility in overcoming endocrine therapy resistance.

As stated above, SETDB1 was identified as a critical mediator of tumor immune evasion, highlighting its potential as a therapeutic target for restoring tumor immune surveillance [28]. We treated MCF‐7 and T47D cells with AP‐SETDB1‐D2 and then incubated them with CD8^+^ T cells isolated from human peripheral blood. The results of lactate dehydrogenase (LDH) detection assays demonstrated that AP‐SETDB1‐D2 also enhances the killing effect of CD8^+^ T cells on breast cancer cells (Figure 7B). This immunomodulatory effect, combined with the direct anti‐tumor activity, highlights the multi‐faceted therapeutic potential of these two PROTACs for breast cancer.

### 2.6 AP‐SETDB1‐S6A and AP‐SETDB1‐D2 Inhibit the Growth of Breast Cancer in Mice

**FIGURE 8:**
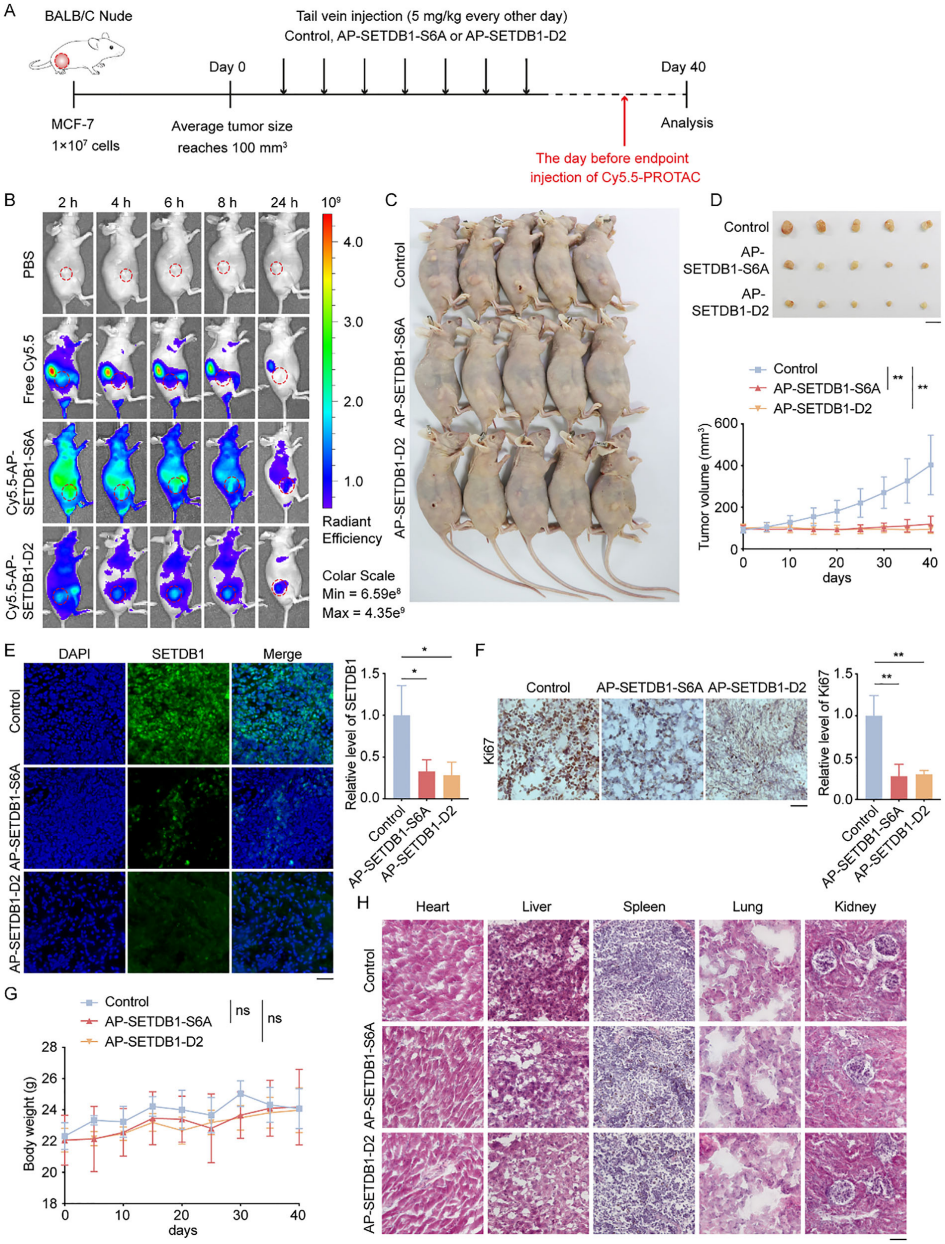
AP‐SETDB1‐S6A and AP‐SETDB1‐D2 inhibit the growth of breast cancer in mice. (A) Schematic diagram of animal experiment procedure. MCF‐7 cells were subcutaneously injected into the right dorsal region of nude mice (BALB/c). Once the tumor volume reached approximately 100 mm^3^, mice were randomly divided into three groups, treated with control PROTAC, AP‐SETDB1‐S6A, and AP‐SETDB1‐D2 at 5 mg/kg by vein injection every other day, respectively. (B) One day before the mice were sacrificed, Cy5.5‐labeled AP‐SETDB1‐S6A and AP‐SETDB1‐D2 were injected into the corresponding group of mice, PBS or Cy5.5 dye only was injected into the control group, via the tail vein. And then living images were captured. (C) Mice were sacrificed and photographed. (D) Tumors were excised and photographed after the sacrifice of the mice. Tumor volumes were monitored at the indicated time. Scale bar, 1 cm. Data are mean ± SD for n = 5; ^**^ *p* < 0.01 (Students’ *t*‐test). (E) Immunofluorescence staining for SETDB1 was performed in subcutaneous tumor tissues, and fluorescence intensity was quantitatively analyzed using ImageJ. Scale bar, 50 µm. (F) Immunohistochemistry staining for Ki‐67 was performed in subcutaneous tumor tissues. Scale bar, 50 µm. For Figures E and F, data are mean ± SD for n = 3; ^*^ *p* < 0.05, ^**^ *p* < 0.01 (Student's *t*‐test). (G) Body weight of mice in the indicated groups was shown. Data are mean ± SD for n = 5; ns, no significant (Student's *t*‐test). (H) Histopathological examination of major organs from mice in the indicated groups.

After establishing the efficacy of AP‐SETDB1‐S6A and AP‐SETDB1‐D2 in cells, we wondered whether they could exhibit their anti‐tumor roles in vivo. MCF‐7 cells were subcutaneously injected into the right dorsal region of nude mice (BALB/c). Once the tumor volume reached approximately 100 mm^3^, mice were randomly divided into three groups, treated with control PROTAC, AP‐SETDB1‐S6A, and AP‐SETDB1‐D2 at 5 mg/kg by vein injection, respectively (Figure 8A). One day before the mice were sacrificed, Cy5.5‐labeled AP‐SETDB1‐S6A and AP‐SETDB1‐D2 were injected into the corresponding group of mice via the tail vein. Live imaging results showed that the Cy5.5 fluorescence signal rapidly accumulated at the tumor tissue sites in Cy5.5‐AP‐SETDB1‐S6A and Cy5.5‐AP‐SETDB1‐D2 treated groups, whereas no significant fluorescence signal aggregation in tumor tissue was observed in the Cy5.5 dye injection group (Figure 8B), indicating the tumor targeting ability of AP‐SETDB1‐S6A and AP‐SETDB1‐D2. As expected, AP‐SETDB1‐S6A and AP‐SETDB1‐D2 significantly attenuated tumor growth compared to the control PROTAC‐treated mice (Figure 8C,D). Immunofluorescent staining of SETDB1 showed that SETDB1 in breast cancer tissues was effectively degraded by AP‐SETDB1‐S6A and AP‐SETDB1‐D2, but not by control PROTAC (Figure 8E). At the meantime, the expression level of Ki‐67, a marker of tumor malignancy, was reduced in AP‐SETDB1‐S6A and AP‐SETDB1‐D2 treated groups (Figure 8F). Importantly, those mice showed no significant weight loss (Figure 8G). Furthermore, histological examinations of liver, spleen, lung, kidney, and heart tissues showed no obvious toxicity (Figure 8H). Collectively, these results indicate that AP‐SETDB1‐S6A and AP‐SETDB1‐D2 are potent SETDB1 degraders, which can directly target breast cancer tissues, penetrate cell membranes to degrade SETDB1, and suppress tumor growth, suggesting their significant therapeutic potential for breast cancer.

## 3 Discussion

SETDB1, a histone methyltransferase responsible for catalyzing H3K9me2 and H3K9me3, has been implicated in breast cancer progression, tamoxifen resistance, and tumor immune evasion. Despite its critical role in oncogenesis, no PROTAC targeting SETDB1 has been developed to date. In this study, we designed aptamer‐based PROTACs by conjugating SETDB1‐specific ssDNA aptamers (identified via SELEX) with AS1411, a nucleolin‐targeting aptamer, and named them AP‐SETDB1‐S6A and AP‐SETDB1‐D2. These two PROTACs can efficiently penetrate breast cancer cells, recruit MDM2 to ubiquitinate SETDB1, and induce its proteasome‐dependent degradation. Importantly, our SETDB1‐targeting PROTACs demonstrated potent anti‐tumor efficacy in vitro and in vivo with minimal cytotoxicity.

TPD has emerged as a groundbreaking therapeutic strategy, particularly for traditionally “undruggable” targets. Compared to conventional inhibitors, TPD offers several advantages. First, TPD can rapidly deplete POI within minutes to hours, minimizing compensatory adaptation. Second, POI can degrade mutated or structurally aberrant POIs, reducing the likelihood of drug resistance. Third, TPD has catalytic, substoichiometric activity. For example, PROTACs can be recycled for multiple rounds of degradation, enhancing potency while lowering effective doses [1]. As important members of TPDs, PROTACs have already shown promise in degrading key oncoproteins, including B‐Raf proto‐oncogene serine/threonine protein kinase (BRAF) V600E in melanoma [40], bromodomain and extraterminal (BET) in prostate cancer [41] and acute myeloid leukemia [42], B‐cell lymphoma extra large (BCL‐XL) in leukemia [43], and estrogen receptor (ER) in breast cancer [44]. Our work extends this paradigm by developing PROTACs against oncogenic protein, SETDB1, which effectively degrades SETDB1 in an MDM2‐ and proteasome‐dependent manner, suppressing breast cancer growth.

The efficacy of PROTACs relies heavily on the specificity of target recognition. Aptamers, with their high specificity and affinity, are increasingly explored as promising recognition moieties for PROTAC design. For instance, ZL216, a chimera of AS1411 and the Von Hippel‐Lindau (VHL) ligand AHPC, degrades nucleolin in breast cancer cells, inhibiting proliferation and migration [45]. Similar aptamer‐PROTAC strategies have been applied to target mutant p53‐R175H [46, 47] and c‐Myc (using the MA9C1 aptamer) [48]. Aptamers have a low molecular weight (8–25 kDa), which facilitates rapid penetration to their target sites in vivo [49]. Additionally, aptamers are typically non‐immunogenic, exhibit high chemical and thermal stability, and can be synthesized and modified inexpensively and scalably [50]. Here, we isolated a high‐affinity SETDB1‐binding aptamer via SELEX and engineered it into dual‐aptamer‐based PROTACs, having all the advantages of aptamers.

A major limitation of conventional PROTACs is their lack of tissue and cell selectivity, often resulting in on‐target, off‐tumor toxicity due to the non‐specific distribution of the PROTAC and widespread expression of E3s in non‐malignant cells. Additionally, cell permeability remains a persistent challenge. Our dual‐aptamer design addresses these issues by exploiting AS1411's ability to selectively target breast cancer cells and facilitate intracellular uptake. Consequently, these PROTACs combine the benefits of aptamers with enhanced tumor selectivity and cell‐penetrating efficiency. However, as nucleic acid‐based constructs, their intracellular stability requires further optimization. In our study, the partial double‐strand PROTAC (AP‐SETDB1‐D2) is more stable and has better SETDB1 degradation effect than the single‐strand PROTAC (AP‐SETDB1‐S6A). We will further explore chemical modifications to improve nuclease resistance and pharmacokinetics in the future. In addition, PROTAC‐based therapies still face important limitations. The formation of ternary complexes is highly context‐dependent and requires precise geometric conformations and spatial orientations, which may result in unpredictable degradation efficiency and off‐target [51]. Therefore, integrating in silico with biochemical approaches to investigate POI–PROTAC–E3 ligase ternary complexes in the future is essential for elucidating their structural activity, cooperativity, and stability [52].

## 4 Experimental Section

### 4.1 Cells and Reagents

MCF‐7 cells were cultured in Dulbecco's modified Eagle's medium (DMEM) (Meilun Biotechnology Co., Ltd., Dalian, China), T47D cells were cultured in Roswell Park Memorial Institute 1640 (RPMI1640) medium (Meilun), and MDA‐MB‐231 cells were cultured in DMEM/F‐12 (1:1) (Meilun), all supplemented with 10% fetal bovine serum (FBS) (Biological Industries, Beit HaEmek, Israel) at 37°C with 5% CO_2_. MCF‐10A cells were cultured in MCF‐10A Cell Complete Medium (DMEM/F12 containing 5% HS, 20 ng/mL EGF, 0.5 µg/mL Hydrocortisone, 10 µg/mL Insulin, 1% NEAA, and 1% P/S) (Procell system Co., Ltd., Wuhan, China) at 37°C with 5% CO_2_. All cells were regularly authenticated by morphological observation and tested for mycoplasma contamination to ensure that the cells were Mycoplasma‐free.

Antibodies and reagents were purchased from the following sources: anti‐SETDB1 (ab107225), anti‐ubiquitin (ab134953), and anti‐MDM2 (ab259265) from Abcam Ltd. (Cambridge, MA, USA); anti‐Ki67 (9027) from Cell Signaling Technology Inc. (Danvers, MA, USA); anti‐β‐actin (AC026) and anti‐NCL (A5904) from ABclonal Technology Co., Ltd. (Wuhan, China); Horseradish peroxidase‐conjugated secondary antibodies (5220–0341 and 5220–0336) from SeraCare Life Sciences Inc. (Milford, MA, USA).

### 4.2 Expression and Purification of His‐Tagged Proteins

The DNA sequence encoding SETDB1 TDDs (amino acids 190–410) was cloned into the pET28a vector. Recombinant proteins were expressed in *E. coli* BL21 (DE3) cells and induced with 0.2 mm isopropyl β‐D‐1‐thiogalactopyranoside (IPTG) at 18°C overnight. Cells were lysed by sonication in lysis buffer (20 mm Tris–HCl, pH 7.5, 400 mm NaCl, and 2 mm β‐mercaptoethanol). Following centrifugation at 12 000 rpm for 30 min, the supernatants were loaded onto Ni‐NTA resin and washed with wash buffer (20 mm Tris–HCl, pH 7.5, 400 mm NaCl, 25 mm imidazole, and 2 mm β‐mercaptoethanol). The recombinant proteins were then eluted with elution buffer (20 mm Tris–HCl, pH 7.5, 400 mm NaCl, 500 mm imidazole, and 5 mm β‐mercaptoethanol). Further purification was performed using a HiTrap Desalting column (GE Healthcare) equilibrated with Phosphate‐Buffered Saline (PBS) supplemented with 5 mm MgCl_2_.

### 4.3 Preparation of Magnetic Bead‐Coupled Proteins

Selection or counter‐selection proteins/peptides were covalently coupled to ‐COOH‐modified magnetic beads (FM2221, Zecheng, Jiangsu, China) via their primary amine groups. Briefly, 50 µL of magnetic beads were washed with 200 µL ddH_2_O, and activated using 50 µL of a 1:1 (v/v) mixture of 0.4 m EDC and 0.1 m NHS for 20 min. The activated beads were then incubated with His‐tagged SETDB1 fragment containing TTDs (50–200 µg/mL in 10 mm NaAc buffer) at room temperature for 1 h with continuous mixing. To block unreacted activation sites, 1 m ethanolamine (HCl, pH 8.5) was added and incubated for 10 min. Finally, the beads were washed three times with PBS containing Mg^2^^+^ (137 mm NaCl, 2.7 mm KCl, 10 mm NaH_2_PO_4_, 2 mm KH_2_PO_4_, 5 mm MgCl_2_, and 0.05% Tween 20).

### 4.4 SELEX Procedure

A single‐stranded DNA (ssDNA) library with a central 36‐nt random sequence flanked by fixed primer regions (5’‐TTCAGCACTCCACGCATAGC and CCTATGCGTGCTACCGTGAA‐3’) was used for SELEX. His‐tagged SETDB1 fragment containing TTDs was used as the target, with His^*^6 peptide serving as the counter‐selection molecule. The SELEX process involved incubating the ssDNA library (1 nmol) with SETDB1‐coated magnetic beads in PBS containing Mg^2^^+^ at room temperature for 1 h. Enriched oligonucleotides were dissociated by boiling at 95°C for 10 min in 200 µL ddH_2_O and subsequently amplified by PCR using both upstream and downstream primer pairs.

To prepare the secondary ssDNA library, PCR amplification was conducted using normal upstream primers and biotin‐labeled downstream primers. The PCR products were incubated with streptavidin magnetic beads (SM017010, Smart‐Lifesciences Biotechnology, Changzhou, China) in 1 m NaCl at room temperature for 30–40 min. DNA double‐strand separation was then achieved by treatment with 40 mm NaOH and 1 m HCl.

In the first and second screening rounds, no negative screening was performed to preserve the diversity of the library sequences. After denaturation, the secondary ssDNA library from the second round was incubated with His^*^6 peptide‐coated magnetic beads for 1 h, followed by incubation with SETDB1‐coated beads. To increase screening pressure, the volume of SETDB1‐coated beads was reduced from 50 to 15 µL, the incubation time was shortened from 1 h to 30 min, and the library input was decreased from 1 nmol to 20 pmol, while maintaining constant negative screening pressure. After several rounds of screening, the enriched DNA library was subjected to high‐throughput sequencing.

After each round of positive screening with SETDB1‐coated magnetic beads, the beads were boiled at 95°C for 10 min in 100 µL ddH_2_O. The supernatant was collected, and 1 µL was used as a template for real‐time quantitative PCR. Data was analyzed to evaluate the SELEX process.

### 4.5 Surface Plasmon Resonance (SPR) Analysis

The dissociation constant (Kd) of aptamers was determined using SPR spectroscopy on a Biacore 8K system (GE Healthcare). All SPR experiments were performed at 24°C with Dulbecco's Phosphate Buffered Saline (DPBS) containing 1.25 mm Mg^2^^+^ as the running buffer. The sensor chip (CM5, Cytiva) was activated by injecting a 50:50 (v/v) mixture of NHS (0.1 m) and EDC (0.4 m) for 5 min. The target protein (50 µg/mL) was diluted in sodium acetate buffer (pH 4.0) and injected into flow cell 2 at a flow rate of 10 µL/min for 5 min, resulting in an immobilization level of approximately 9753.2 response units (RU). Meanwhile, the control protein (5 µg/mL) was diluted in sodium acetate buffer (pH 5.0) and immobilized on flow cell 1 under the same conditions, yielding a surface with 480.2 RU. This reference surface was used to subtract bulk refractive index shifts and non‐specific binding signals. The sensor chip was then blocked with 1 m ethanolamine‐HCl (pH 8.5) for 2 min.

Aptamers were prepared at an initial concentration of 500 nm in DPBS containing 1.25 mm Mg^2^^+^. All buffers were filtered and degassed prior to use. Binding assays were conducted by injecting aptamers at a series of concentrations over the sensor chip at a flow rate of 30 µL/min. The association and dissociation phases were monitored for 3 min each. PBS and 1 m NaCl were used as the running and regeneration buffers, respectively. The resulting sensorgrams, representing the association and dissociation of the protein‐aptamer complex, were processed by subtracting the background signals from flow cell 1. The equilibrium dissociation constant (Kd) was calculated by globally fitting the data to a 1:1 Langmuir binding model using the Biacore Evaluation Software (Cytiva).

### 4.6 Isothermal Titration Calorimetry (ITC)

ITC experiments were conducted at 25°C using a MicroCal PEAQ‐ITC instrument (Malvern Panalytical, Worcestershire, UK). Proteins and aptamers/PROTACs were prepared in an ITC buffer identical to the screening buffer (137 mm NaCl, 2.7 mm KCl, 10 mm NaH_2_PO_4_, 2 mm KH_2_PO_4_, and 5 mm MgCl_2_). During the titration, 350 µL SETDB1 protein solution (protein concentration: 10 µm) was loaded into the titration cell, and 3 µL aptamer/PROTAC solution (100 µm) (with the exception of the first injection of 0.4 µL) was titrated into the cell, with a 120‐s interval and a reference power of 5 µCal/s. The titration data were analyzed using the one‐site binding model from MicroCal PEAQ‐ITC Analysis Software (V1.30).

### 4.7 Synthesis of an Aptamer‐Based Partial dsDNA PROTAC

Individual aptamers synthesized were first denatured at 95°C for 10 min in PBS buffer containing 5 mm MgCl_2_, pH 7.5, followed by gradual cooling to 4°C. The two partial complementary aptamers were then mixed at a molar ratio of 1:1 and incubated at 4°C for 2 h to promote self‐assembly. The successful formation of AP‐SETDB1‐Dn was confirmed by native polyacrylamide gel electrophoresis (PAGE).

### 4.8 Streptavidin Pulldown Assays

MCF‐7 cells were lysed in NETN lysis buffer (20 mm Tris‐Cl, pH 8.0, 100 mm NaCl, 1 mm EDTA, and 0.5% NP‐40) supplemented with a protease inhibitor cocktail and PMSF. After centrifugation, the supernatants were collected, and protein concentrations were determined using a NanoDrop 2000 spectrophotometer (Thermo Scientific). The cell lysate (1 mg) was then incubated with 10 µg biotin‐aptamer, biotin‐PROTAC, or biotin‐control at 4°C overnight, followed by further incubation with 50 µL streptavidin MagPoly beads (Smart‐Lifesciences) at 4°C for 2–4 h. The beads were washed three times with washing buffer (20 mm Tris‐Cl, pH 8.0, 100 mm NaCl, 1 mM EDTA, and 0.1% NP‐40). After washing, the beads were resuspended in 1 × SDS–PAGE loading buffer and boiled at 98°C for 10 min. The protein samples were then subjected to Western blotting.

### 4.9 RNA Interference

Small interfering RNA (siRNA) targeting NCL (siNCL: GGAUGACGACGACGACGAAGATT) or a negative control siRNA (UUCUCCGAACGUGUCACGUTT) was transfected into MCF‐7 cells using Lipofectamine RNAiMAX Transfection Reagent (13778150, Thermo Fisher Scientific Inc.), according to the manufacturer's instructions. For lentiviral production, an effective siRNA sequence targeting MDM2 (siMDM2: GAUUCCAGAGAGUCAUGUGTT) or a control siRNA sequence (CCUAAGGUUAAGUCGCCCUCG) was cloned into the pLKO vector. The resulting lentiviral constructs were co‐transfected into HEK‐293FT cells along with packaging plasmids (PAX2 and pMD2.G) using polyethyleneimine (PEI; 23966, Polysciences, PA, USA). Infectious lentiviruses were collected and filtered through a 0.45 µm pore‐size membrane at 24 and 48 h post‐transfection. MCF‐7 cells were then infected with the lentiviruses in the presence of polybrene (1 µg/mL), followed by puromycin selection (1.5 µg/mL) to establish stable knockdown cells.

### 4.10 MTT Assay

Cell viability was assessed using the 3‐(4, 5‐dimethylthiazol‐2‐yl)‐2, 5‐diphenyl‐2H‐tetrazolium bromide (MTT) assay. Cells were seeded in 96‐well plates at approximately 2000 cells per well and incubated with Veh (PBS), AS1411+C11, AP‐SETDB1‐S6A, control PROTAC, or AP‐SETDB1‐D2 for 0, 2, 4, 6, or 8 days. The culture medium containing corresponding PROTACs was refreshed every two days. At each time point, MTT solution (M8180, Solarbio, Beijing, China, final concentration is 0.5 mg/mL) was replaced in each well for a 4‐hour incubation. After removal of the MTT solution, 110 µL dimethyl sulfoxide (DMSO) was added to dissolve the formazan product, and the absorbance was spectrophotometrically measured at 490 nm using a microplate reader.

### 4.11 Colony Formation Assay

MCF‐7 and T47D cells (800 and 400 cells per well, respectively) were seeded into wells of a six‐well plate. The culture medium containing control or SETDB1 PROTAC was refreshed every 2 days. After 2 weeks, the medium was removed, and cells were washed twice with PBS and then fixed with 4% paraformaldehyde for 15 min, followed by staining with 0.1% crystal violet solution (Solarbio) for 15 min. Images of the colonies were captured, and the colony numbers were counted using ImageJ software.

### 4.12 EdU‐Incorporation Assay

The EdU‐incorporation assay was performed using BeyoClick EdU‐488 (C0071S, Beyotime, Shanghai, China) according to the manufacturer's instructions. Briefly, cells were seeded in 96‐well plates at a density of 6000 cells per well and incubated with control or SETDB1 PROTACs. After 48 h of incubation, cells were cultured with EdU solution (EdU final concentration is 10 µm) for 2 h and then fixed with 4% paraformaldehyde for 15 min. Following rinsing with washing buffer (3% bovine serum albumin in PBS), cells were permeabilized with 0.3% Triton X‐100 in PBS for 10 min and treated with 100 µL of click reaction solution for 30 min. Subsequently, Hoechst 33342 was added to stain the cell nuclei for 10 min in the dark, and images were captured using a fluorescence microscope.

### 4.13 Wound Healing Assay

Cell migration capability was measured using the wound healing assay as described previously [53]. Cells treated with control or SETDB1 PROTACs were cultured in six‐well plates to reach 100% confluency. A pipette tip was used to create a wound line across the surface of the plates. Cells were washed with PBS three times and cultured in medium without FBS to inhibit proliferation. At 0, 24, and 48 h after the scratch, the width of wounds was imaged using a light microscope and analyzed using ImageJ software.

### 4.14 Transwell Assay

Transwell assays were performed as previously described [54]. T47D and MDA‐MB‐231 cells were incubated with control or SETDB1 PROTACs for 48 h. About 1 × 10^4^ cells were suspended in 200 µL of medium without serum and placed in an upper chamber precoated with 100 µL Matrigel (BD Biosciences) solution (8 µL Matrigel mixed with 92 µL DMEM). The lower chamber was filled with 600 µL of medium containing 20% FBS. After 24 h of incubation, the cells that invaded through the matrigel were fixed and stained with 0.1% crystal violet. Images were captured using a microscope.

### 4.15 Isolation of Peripheral Blood Mononuclear Cells (PBMCs) and CD8^+^ T Cells

PBMCs were isolated from human peripheral blood using density gradient centrifugation with lymphocyte separation solution (LTS1077, HaoYang, China). Briefly, blood samples were collected using EDTA‐K_2_ as an anticoagulant. The lymphocyte separation solution was preloaded into sterile centrifuge tubes, followed by the gentle addition of an equal volume of fresh blood. The samples were centrifuged at 600 g for 30 min at 20°C. The intermediate, opaque white layer containing lymphocytes was carefully aspirated and transferred to a new sterile centrifuge tube. PBS was added at three times the volume for dilution, followed by centrifugation at 300 g for 10 min. The cells were washed twice with PBS, and the final pellet was designated as PBMCs. The cells were counted, and a suspension was prepared at a concentration of 1 × 10^8^ cells/mL.

CD8^+^ T cells were isolated from peripheral blood mononuclear cells (PBMCs) using ImunoSep Human CD8^+^ Cell Positive Selection Kit (712805, JinZhun, China) according to the manufacturer's instructions. Based on the volume of the prepared single‐cell suspension, 10 µL of Sorting Reagent A was added to 100 µL of the cell suspension, thoroughly mixed, and incubated at room temperature for 10 min. The cells were then centrifuged and resuspended to their original volume. Subsequently, 10 µL of Sorting Reagent B was added, followed by mixing and incubation under the same conditions. After incubation, magnetic separation was performed to isolate CD8^+^ T cells. The isolated cells were resuspended in RPMI 1640 culture medium and cultured with 2 µg/mL CD3 and CD28 for 24 h. IL‐2 (10 ng/mL) was then added, and the culture was continued for an additional 48 h to complete in vitro activation. The activated CD8^+^ T cells were subsequently used for the T cell killing assay.

### 4.16 CD8^+^ T Cell‐Mediated Tumor Cell Killing Assay

MCF‐7 and T47D cells were incubated with SETDB1 PROTAC or control PROTAC for 24 h. Activated CD8^+^ T cells (1 × 10^4^ cells/well) were seeded into 96‐well plates at an effector‐to‐target ratio of 10:1 and co‐cultured with the pre‐treated tumor cells for 48 h. Following incubation, lactate dehydrogenase (LDH) released in the supernatant was measured using a one‐step assay using the LDH Cytotoxicity Assay Kit (MA0649, MeiLun, China) according to the manufacturer's instructions.

### 4.17 Animal Experiments

Animal studies were approved by the Institutional Animal Care and Use Committees of Tianjin Medical University (TMUaMEC 2022008). For cell line‐derived xenografts models, 1 × 10^7^ MCF‐7 cells were suspended in 50 µL PBS, mixed with Matrigel (1:1 volume), and subcutaneously injected into the right flank of the female athymic nude mice (BALB/c; 5‐6 weeks of age; 5 mice per group) (Charles River Laboratories, Wilmington, MA, USA). Once the tumor volume reached approximately 100 mm^3^, mice were randomly divided into three groups and treated with AP‐SETDB1‐S6A, AP‐SETDB1‐D2, or control PROTAC (5 mg/kg) via tail vein injection every other day. The tumor sizes were measured with a Vernier caliper and calculated using the formula: V = π/6 × length × width^2^. After 40 days, the mice were euthanized, and the tumors were dissected out and weighed.

For the in vivo imaging, at one day before the mice were euthanized, mice in the AP‐SETDB1‐S6A and AP‐SETDB1‐D2 groups were intravenously injected with Cy5.5‐labelled AP‐SETDB1‐S6A (5 mg/kg) and AP‐SETDB1‐D2, respectively. Control mice were injected with PBS or free Cy5.5. At 2, 4, 6, 8, and 24 h post‐injection, the mice were anesthetized with isoflurane gas and imaged using IVIS SPECTRUM system (PerkinElmer Instruments Co., Ltd., Massachusetts, USA).

### 4.18 Immunohistochemistry Analysis

Xenograft tumor samples retrieved from mice were embedded in an Optimal Cutting Temperature (OCT) compound (BL1674A; Biosharp, Beijing, China) and rapidly frozen at −80°C. The frozen tissues were subsequently sectioned into consecutive 8 µm slices. Prior to immunostaining, tissue sections were treated with 3% hydrogen peroxide for 10 min in the dark to suppress endogenous peroxidase activity, followed by blocking with PBST (PBS containing 0.1% Triton X‐100) supplemented with 10% goat serum for 1 h to prevent nonspecific binding. The sections were then incubated overnight at 4°C with primary antibodies and then incubated at room temperature with horseradish peroxidase (HRP)‐conjugated secondary antibodies for 1 h. Signal visualization was achieved using a 3,3′‐diaminobenzidine (DAB) substrate kit (ZLI‐9017; ZSGB‐BIO, Beijing, China), and the slides were counterstained with hematoxylin (G1120; Solarbio). Images were acquired using a light microscope (Axio Imager M2, ZEISS, Germany).

### 4.19 Statistical Analysis

All experimental data are presented as mean ± SD or mean ± SEM (mean ± SEM was used only for IC50 assays in Figure 7A), based on at least three (n = 3) biological replicates. Statistical analyses were conducted using IBM SPSS Statistics software (version 19.0). Comparisons between two groups were performed using Student's *t*‐test, whereas multiple group comparisons were assessed using one‐way ANOVA followed by either Bonferroni or Tamhane's T2 post‐hoc tests to evaluate statistical differences.

## Author Contributions

C.X. conceived and supervised the project, wrote and revised the manuscript. Y.G., Y.L., and S.H. performed most of the experiments. Y.G. wrote the manuscript. C.L. and Y.O. performed some experiments. B.L. checked the original data.

## Conflicts of Interest

The authors declare no conflicts of interest.

## Supporting information




**Supporting File**: advs73716‐sup‐0001‐SuppMat.docx.

## Data Availability

The data that support the findings of this study are available from the corresponding author upon reasonable request.
