# Supplementary material for: Dual Aptamers‐Based SETDB1 PROTACs as Effective Anti‐Tumor Strategies for Breast Cancer
Source: Adv Sci (Weinh). 2026 Jan 7;13(18):e21159. doi: 10.1002/advs.202521159 (PMC13042445; doi:10.1002/advs.202521159)
Supplement: Supplementary file 1 — Supporting File: advs73716‐sup‐0001‐SuppMat.docx. [file ADVS-13-e21159-s001.docx]

**Supporting Information**

**Supplementary Figures and Table**


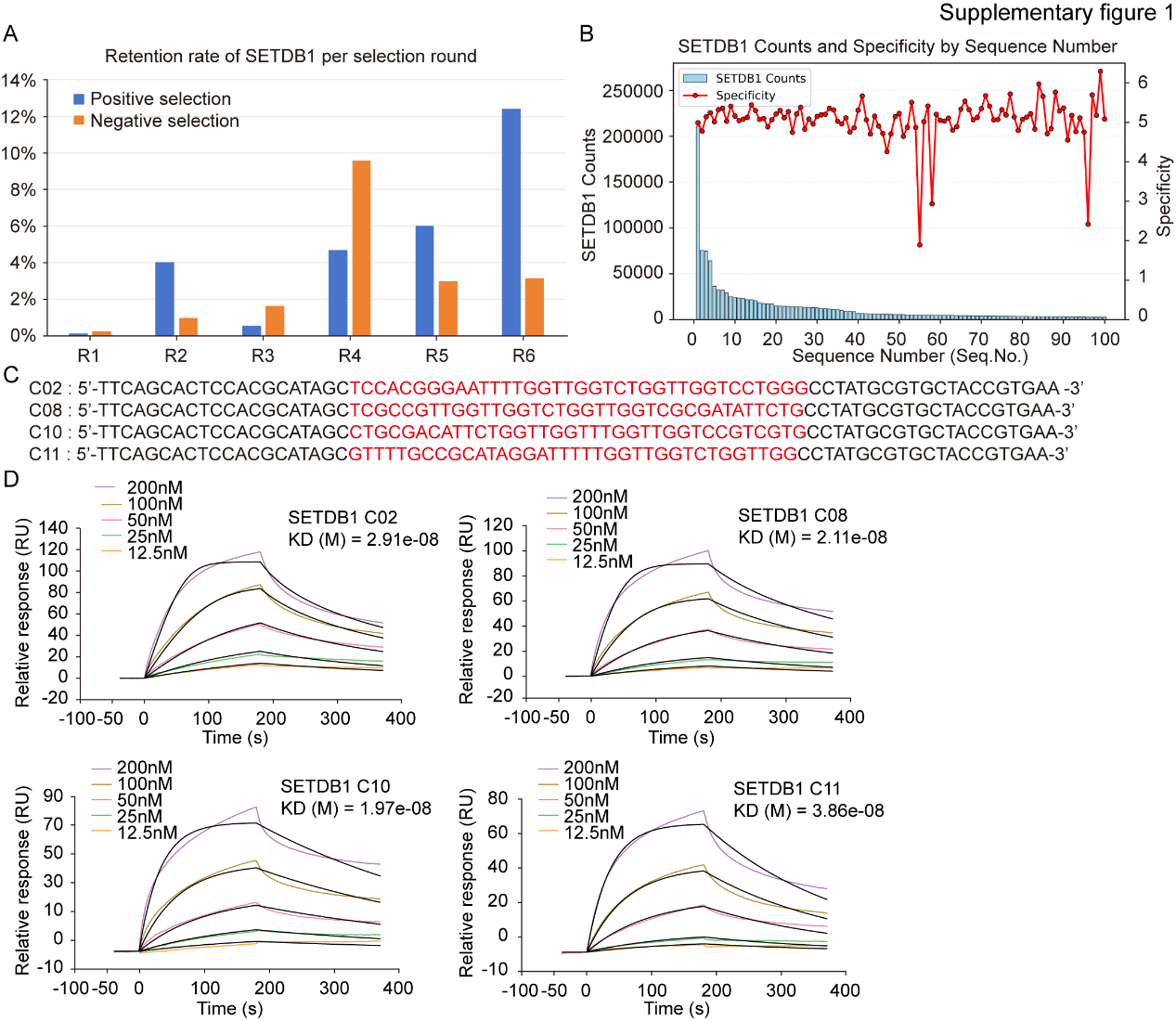


**Supplementary Figure 1. In vitro selection of ssDNA aptamers for SETDB1 protein.**

**A.** Positive and negative retention rates of the enriched DNA pool using RT-qPCR across rounds 1 to 6. **B.** Following six rounds of SELEX, high-throughput sequencing was performed, and the specificity coefficients of the top 100 sequences were analyzed. **C.** The sequence of candidate aptamers (C02, C08, C10, C11) containing linkers. **D.** SPR assays were performed to assess the binding affinity of SETDB1 TTDs with aptamers (C02, C08, C10, and C11).


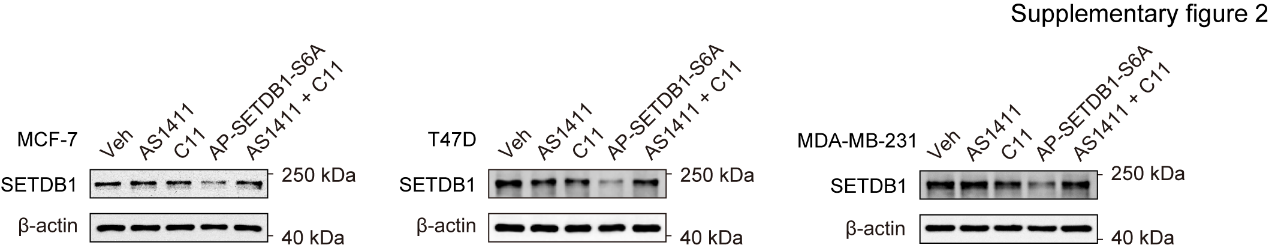


**Supplementary Figure 2.** **SETDB1 degradation requires the intact AP-SETDB1-S6A.**

The protein levels of SETDB1 were analyzed by Western blotting in MCF-7, T47D, and MDA-MB-231 cells after a 24-hours incubation with PBS, 1 μM of AS1411, C11, AP-SETDB1-S6A, or a mixture of AS1411 and C11.

**Supplementary table 1. Oligonucleotides used in this study.**

| Name | Sequences (5' to 3') |
| --- | --- |
| C2-nolinker | TCCACGGGAATTTTGGTTGGTCTGGTTGGTCCTG  GG |
| C8-nolinker | TCGCCGTTGGTTGGTCTGGTTGGTCGCGATATTC  TG |
| C10-nolinker | CTGCGACATTCTGGTTGGTTTGGTTGGTCCGTCG  TG |
| C11-nolinker | GTTTTGCCGCATAGGATTTTTGGTTGGTCTGGTT  GG |
| C11-1 | TTTGCCGCATAGGATTTTTGGTTGGTCTGGTT |
| C11-2 | TGCCGCATAGGATTTTTGGTTGGTCTGG |
| C11-3 | CCGCATAGGATTTTTGGTTGGTCT |
| AS1411 | GGTGGTGGTGGTTGTGGTGGTGGTGG |
| C11 | GTTTTGCCGCATAGGATTTTTGGTTGGTCTGGTT  GG |
| RS | AATTTAGTACTCTGTTCTTTCCTTTATTCTAATTGT  TTAA |
| AP-SETDB1-S6A | GGTGGTGGTGGTTGTGGTGGTGGTGGAAAAAAG  TTTTGCCGCATAGGATTTTTGGTTGGTCTGGTTGG |
| AP-SETDB1-S10A | GGTGGTGGTGGTTGTGGTGGTGGTGGAAAAAAA  AAAGTTTTGCCGCATAGGATTTTTGGTTGGTCTG  GTTGG |
| AP-SETDB1-S15A | GGTGGTGGTGGTTGTGGTGGTGGTGGAAAAAAA  AAAAAAAAGTTTTGCCGCATAGGATTTTTGGTTG  GTCTGGTTGG |
| AP-SETDB1-2-S6A | GGTGGTGGTGGTTGTGGTGGTGGTGGAAAAAA  TGCCGCATAGGATTTTTGGTTGGTCTGG |
| C11-linker1 | TGCCGCATAGGATTTTTGGTTGGTCTGGTTTCGG  GATCCC |
| C11-linker2 | TGCCGCATAGGATTTTTGGTTGGTCTGGTTGGCC  GGCGCT |
| C11-linker3 | TGCCGCATAGGATTTTTGGTTGGTCTGGTTGTTG  CCCGTG |
| AS1411-linker1 | GGTGGTGGTGGTTGTGGTGGTGGTGGAATGGGA  TCCCGA |
| AS1411-linker2 | GGTGGTGGTGGTTGTGGTGGTGGTGGAATAGCG  CCGGCC |
| AS1411-linker3 | GGTGGTGGTGGTTGTGGTGGTGGTGGAATCACG  GGCAAC |
| RS-linker2 | AATTTAGTACTCTGTTCTTTCCTTTATTCTAATTGTT  TAATTGGCCGGCGCT |
| Control | RS-linker2 + AS1411-linker2 |
| AP-SETDB1-D1 | C11-linker1 + AS1411-linker1 |
| AP-SETDB1-D2 | C11-linker2 + AS1411-linker2 |
| AP-SETDB1-D3 | C11-linker3 + AS1411-linker3 |
